# Sensorimotor conflict tests in an immersive virtual environment reveal subclinical impairments in mild traumatic brain injury

**DOI:** 10.1038/s41598-020-71611-9

**Published:** 2020-09-08

**Authors:** Hrishikesh M. Rao, Tanya Talkar, Gregory Ciccarelli, Michael Nolan, Anne O’Brien, Gloria Vergara-Diaz, Delsey Sherrill, Ross Zafonte, Jeffrey S. Palmer, Thomas F. Quatieri, Ryan J. McKindles, Paolo Bonato, Adam C. Lammert

**Affiliations:** 1 Human Health & Performance Systems, MIT Lincoln Laboratory, Lexington, MA USA; 2 Motion Analysis Laboratory Spaulding Rehabilitation Hospital, Boston, MA USA; 3 Department of Physical Medicine and Rehabilitation, Spaulding Rehabilitation Hospital, Massachusetts General Hospital, Boston, MA USA; 4 Brigham and Women’s Hospital, Harvard Medical School, Boston, MA USA; 5 Department of Biomedical Engineering, Worcester Polytechnic Institute, Worcester, MA USA; 6 Speech and Hearing Bioscience and Technology, Harvard Medical School, Boston, MA 02115 USA

**Keywords:** Diagnostic markers, Brain injuries, Translational research

## Abstract

Current clinical tests lack the sensitivity needed for detecting subtle balance impairments associated with mild traumatic brain injury (mTBI). Patient-reported symptoms can be significant and have a huge impact on daily life, but impairments may remain undetected or poorly quantified using clinical measures. Our central hypothesis was that provocative sensorimotor perturbations, delivered in a highly instrumented, immersive virtual environment, would challenge sensory subsystems recruited for balance through conflicting multi-sensory evidence, and therefore reveal that not all subsystems are performing optimally. The results show that, as compared to standard clinical tests, the provocative perturbations illuminate balance impairments in subjects who have had mild traumatic brain injuries. Perturbations delivered while subjects were walking provided greater discriminability (average accuracy ≈ 0.90) than those delivered during standing (average accuracy ≈ 0.65) between mTBI subjects and healthy controls. Of the categories of features extracted to characterize balance, the lower limb accelerometry-based metrics proved to be most informative. Further, in response to perturbations, subjects with an mTBI utilized hip strategies more than ankle strategies to prevent loss of balance and also showed less variability in gait patterns. We have shown that sensorimotor conflicts illuminate otherwise-hidden balance impairments, which can be used to increase the sensitivity of current clinical procedures. This augmentation is vital in order to robustly detect the presence of balance impairments after mTBI and potentially define a phenotype of balance dysfunction that enhances risk of injury.

## Introduction

Each year, approximately 1.7 million people in the United States (US) sustain a mild traumatic brain injury (mTBI), or concussive event, primarily the result of a fall, car accident, or sport injury^1,2^. An individual with mTBI may present with a combination of sensorimotor impairments (e.g., deficits in vestibular, visual, or proprioceptive function) with associated dizziness and balance problems^3^. Between 15 and 40% of mTBI patients experience incomplete recoveries^4,5^, or may provide subjective reports of decreased balance confidence despite a clinically-determined complete recovery. These physical symptoms are augmented by difficulties performing cognitive tasks, such as focused attention and impaired memory recall^6^. In addition, those with recent mTBI or concussion appear to be at an enhanced risk for musculoskeletal injury, thus it may be critical to define subtle balance dysfunction^7^.

Theoretical understanding of the etiology of these lingering difficulties is an active area of research, and includes fundamental debate over, e.g., whether they are physiological or psychological in nature^8^. Whatever their cause, the immediate problem facing clinicians is to perform assessment, in order to determine a course of treatment, within a population that is inherently heterogeneous in terms of injury type, time since injury and history of treatment. Despite the wide heterogeneity, the incidence of an mTBI may be enough to cause subtle changes in gait strategy that can be detected through precise measurement. Current clinical tests aimed at assessing mobility and cognitive function are not sensitive enough to uncover subtle sensorimotor impairments associated with mTBI diagnoses, especially long after injury^9^. First, the symptoms reported are often subtle and their impact on the performance of everyday activities, although significant, is often hidden because the person has adapted to perform daily activities even in their altered state. Second, most of the current assessment strategies focus on simple movements or on cognitive tasks, rather than dual task scenarios or other combined conditions. Consequently, those strategies are not capable of measuring an individual’s change within the bounds of a normative population. Even measures such as the Bess Test, which have been recommended for assessment after mTBI, have been shown to be not sensitive after 3–5 days of injury^10^. Third, only a few tests push subjects into challenging sensorimotor control situations that elicit underlying deficits^11^. This is especially important if the nature of the impairment affects only certain types of sensorimotor control, such as ones that require closed-loop interaction between sensory modalities. Therefore, there is a pressing need to go beyond standard clinical assessments to uncover otherwise hidden impairments and to understand how the wide array of deficits observed in mTBI impact static and dynamic balance.

Perturbing sensorimotor systems can improve assessment sensitivity, and provide insights into the specific nature of impairments^12^. For example, several widely-used clinical assessments ask patients to stand with eyes closed, stand on foam pads, or stay upright in the face of force applied (usually manually) to the body. These perturbations can be viewed as simple ways of allowing clinicians to manipulate sensory feedback and sensorimotor integration. The advantages afforded by perturbations served as partial inspiration for posturography systems that apply perturbations and capture patient responses in a more objective and quantifiable manner. Perhaps the most notable such system is the NeuroCom system (NeuroCom International, Inc, Clackamas, OR) and its implementation of the Sensory Organization Test. Recent advances in virtual environment technology have enabled the implementation of a broader range of provocative tests^13–15^, which variously implement perturbations in a more ecologically valid context^16^, or take advantage of the greater control and variety of perturbations afforded by immersive environments^13–15^. More advanced systems, such as the one used in this study, contain the necessary tools for implementing perturbations during gait, indeed even synchronized with specific parts of the gait cycle. The most notable inroads have been made by using immersive environments, and the broad range of perturbations they allow, to challenge patients balance as part of rehabilitation programs^17,18^.

Unique to the present work is the use of immersive sensorimotor perturbations to detect subtle balance impairments following an mTBI. Further, these subjects have been otherwise deemed “normal” by standard clinical tests, yet they subjectively report low confidence in balance. To enable detection of nuanced differences between populations, a novel battery of perturbations were used independently and in concert, as well as during standing conditions or timed to specific parts of the gait cycle.

**Figure 1:**
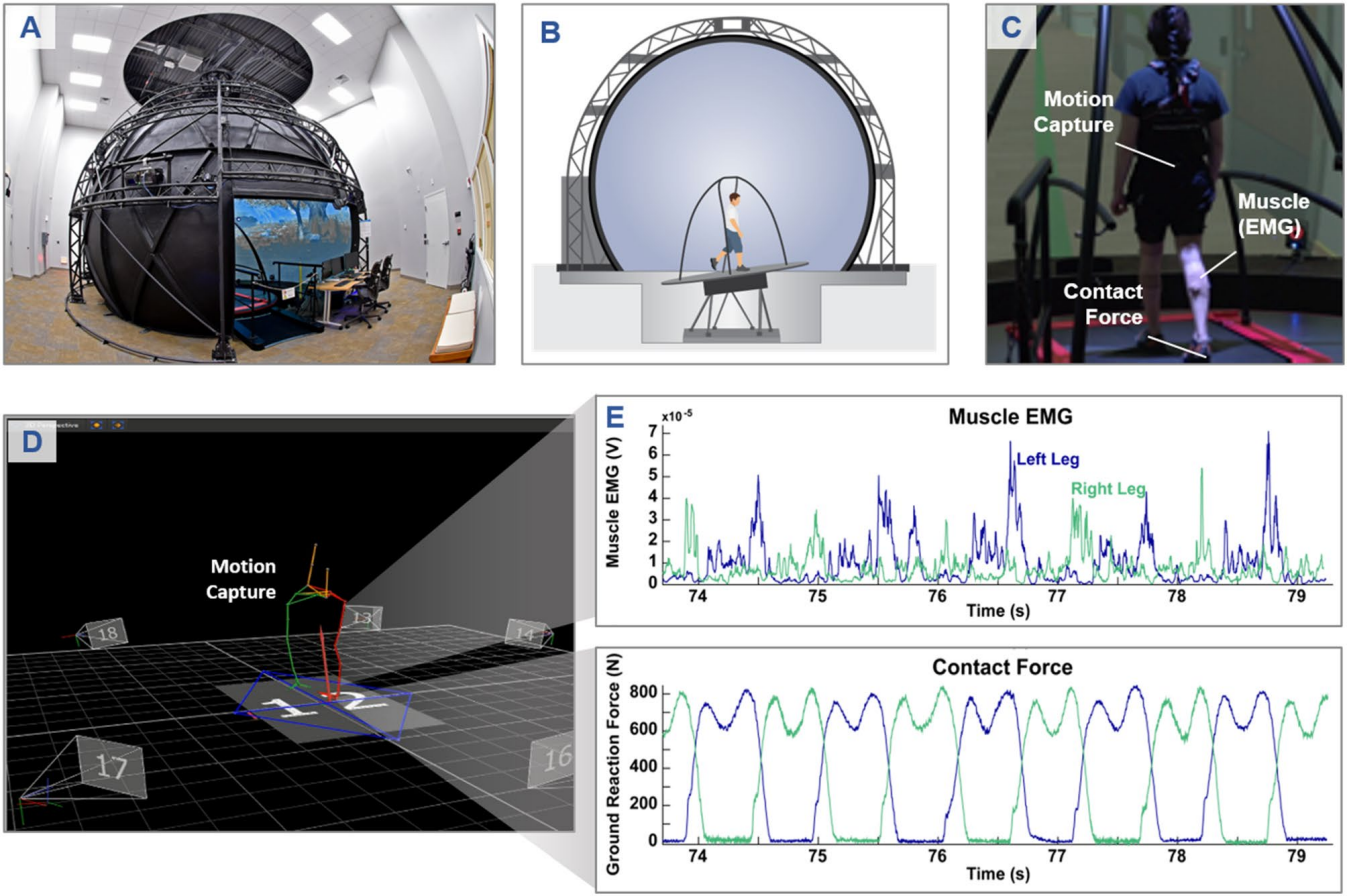
Instrumented immersive virtual environment. (**A**) Photograph of the MIT Lincoln Laboratory CAREN system. (**B**) A cross-sectional schematic of the CAREN system illustrating its capabilities for delivering visual and physical perturbations to individual subjects. (**C**) Subject walking on the treadmill in the virtual hospital hallway, undergoing a platform rotation perturbation. (**D**) Motion capture data of the subject collected concurrently with the perturbation, and (**E**) a subset of the signals collected during the experiment using the CAREN instrumentation.

At the core of the experimentation detailed in the present work is an instrumented, virtual environment capable of delivering immersive perturbations while measuring the physiology of the subject. Experiments were conducted in a Computer-Assissted Rehabilitation ENvironment (CAREN) system (Fig. 1A) at the MIT Lincoln Laboratory (MIT LL) Sensorimotor Technology Realization in Immersive Virtual Environments (STRIVE) Center^19, 20^. This system includes an instrumented, dual-belt treadmill mounted on a six degree of freedom motion base (Fig. 1B). The motion base is centered in a 7-m diameter dome that doubles as a 360° projection screen that immerses the subject in a virtual world (Fig. 1B). In addition to precise stimulus delivery, the system enables precision motion capture and collection of physiological data using wearable and instrumented sensors (Fig. 1C–E).

Our primary hypothesis is that sensory conflicts, induced through provocative sensorimotor perturbations during dynamic balance tasks, will illuminate nuanced differences between healthy control subjects and those who have persistent post-concussive syndrome. The present work tests the notion that advanced sensing, signal processing, and statistical modeling can illuminate subtle impairments stemming from mTBI, which are otherwise hidden in standard clinical tests. This notion is tested, in perhaps the most difficult situation, wherein impairments may remain beyond the acute phase of injury. Given that a clinical “gold standard” for detecting sensorimotor impairment does not exist, this work focuses on clinical diagnoses as a proxy for TBI-induced impairment. The guiding concept is that, if specific perturbation tests and gait-related features can detect subtle injury-related impairments, then those features should be able to classify individuals by clinical diagnosis (i.e., patient with mTBI vs. healthy control). Therefore, the focus is on the degree to which it is possible to discriminate individuals with a clinical diagnosis of mTBI from healthy controls using a range of sensorimotor provocative tests and detailed behavioral features. Relatedly, the question of which features and tests provide good classification is addressed, as well as how those features relate to self-reported balance confidence, which could aid in treatment planning.

## Results

Twenty-one subjects participated in this study. Ten were healthy controls and eleven were subjects with mTBI. The recruitment of the healthy controls were targeted to match the gender, age, height, and weight distribution of the mTBI subject pool (Table S1). Six out of the 11 subjects with mTBI were still being followed by a physician according to their treatment plan. Those subjects were referred for this study by their physicians while other subjects were recruited through word-of-mouth and advertistments. The mTBI subjects included five females, had a mean age of 39.1 years (SD = 17.18), height of 1.73 m. (SD = 0.1), and weight of 78.47 kgs. (SD = 22.2). The control group included four females, had a mean age of 32.4 years (SD = 14.0), height of 1.73 m. (SD = 0.08), and weight of 72.8 kgs. (SD = 17.42). Clinical assessments showed that all subjects had normal lower extremity range of motion, strength, proprioception and were negative for benign paroxysmal positional vertigo (BPPV) by Dix-Hallpike maneuver.

### Sensitivity of clinical tests

**Figure 2:**
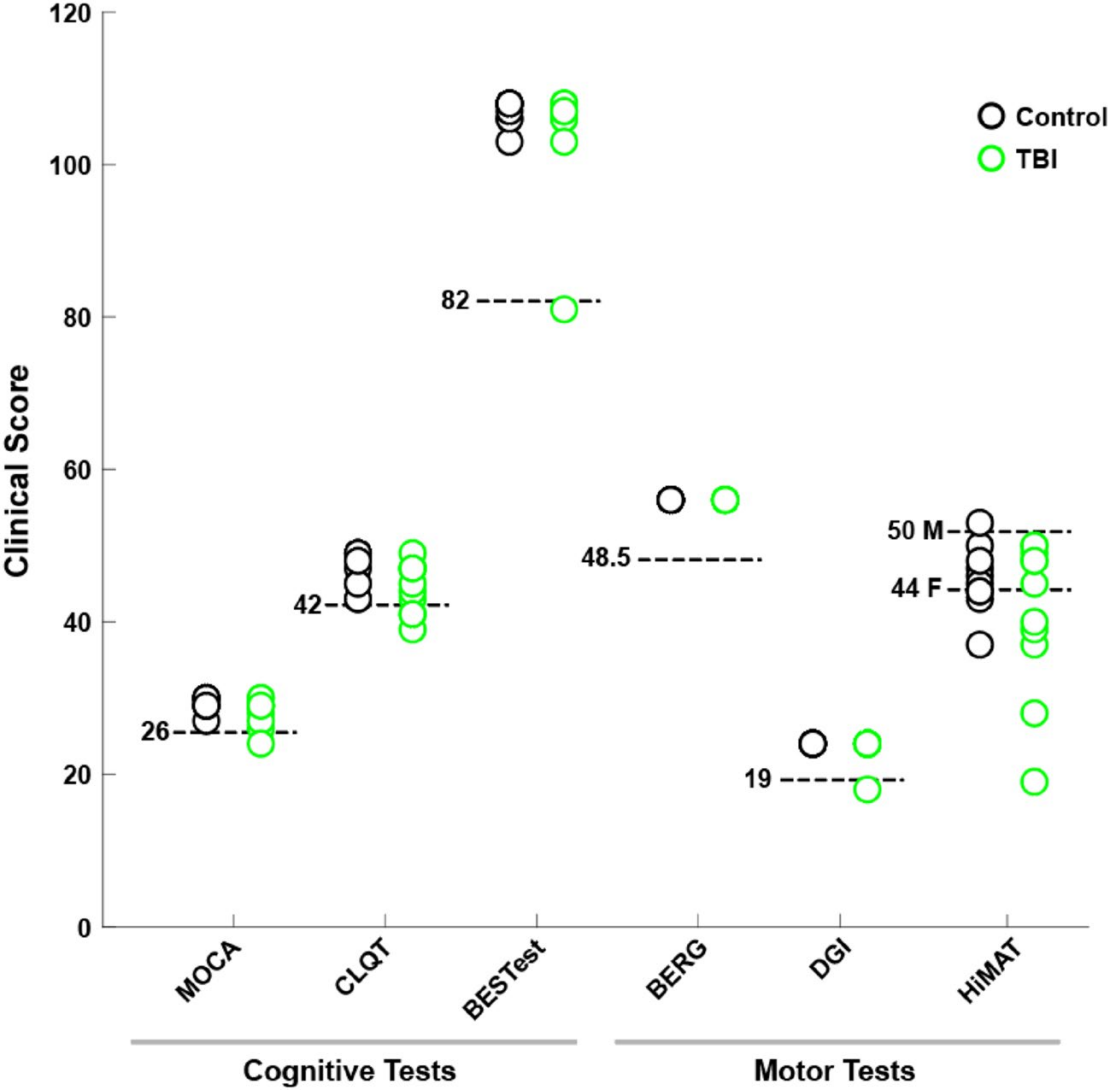
Clinical test scores. Each data point represents the score on the clinical test for subjects in the control group (black open circles) or subject in the mTBI group (green open circles). Within each test category, a clinically-relevant threshold for normative scores (dashed lines) is listed, as determined in literature. In the case of the HiMAT test, the thresholds are different for males (*M*) and females (*F*). Scores below the threshold would indicate impairment in that category.

A battery of standard clinical tests was administered to subjects by an experienced clinician immediately prior to the novel provocative testing. The clinical tests themselves consisted of cognitive- and motor-focused assessments (Fig. 2), and included: Montreal Cognitive Assessment (MOCA), Cognitive Linguistic Quick Test (CLQT), Balance Evaluation Systems Test (BESTest), Berg Balance Scale, Dynamic Gait Index (DGI) and the High-Level Mobility Assessment Tool (HiMAT). This battery yielded little capability for discriminating between the healthy control and the mTBI population. The tests were insensitive as the majority of participants (100% of controls, 89% of mTBI subjects), whether in the healthy control or mTBI group, scored above the thresholds cited (Fig. 2, dashed lines), meaning no impairment. In some cases, e.g. HiMAT, the tests were poorly specific and the majority of subjects scored below the cited thresholds (HiMAT: 50% of controls, 82% of mTBI subjects), indicating impariments the majority of subjects even in the control group. However the normative values for the HiMAT are for 18–25 years old and our sample contained several subjects who were above this range. Therefore, these standard cognitive and sensorimotor clinical tests do not provide the sensitivity or specificity needed to provide accurate detections of mTBI. Yet, through self-reports to their physicians and the Activities-Specific Balance Confidence (ABC) scale (see Table S1), these mTBI subjects still continue to report lingering balance impairiments sustained due to their brain injuries.

### Predictive capability of sensorimotor perturbations

While in the CAREN system, subjects were asked either to stand stationary or to walk down a simulated hallway at a comfortable pace. A variety of sensory conflict situations were created by perturbations to either the visual scene or the platform configuration. Platform perturbations were delivered in one of two ways. The platform could be slowly (over 5 s) rotated downward, with the aim of causing the subject's entire body to rotate in space (Fig. 1B). Alternatively, the platform would be rapidly (< 1 s) translated in the forward direction of the subject, with the aim of moving the subject's lower limbs relative to the upper body. The visual scene could also be perturbed simultaneously in a congruent manner (i.e., movement in the same direction as the platform) or incongruent manner (i.e., movement in opposite directions as the platform). The permutation of modalities (visual or platform), combination (visual/platform only or together), motion (translation or rotation), and congruence (congruent or incongruent) yielded eight different types of perturbations.

Lower limb motion capture, accelerometry, and electromyography (EMG) data were collected as subjects performed the experiment. From these, four categories of features were computed – spatiotemporal (e.g., stride length), kinematic (e.g., joint angles), accelerations, and EMG features. To isolate the influence of the perturbation on changes in balance while minimizing natural variability in individual differences, features were computed in the period after the perturbation (5 s window) and normalized by the features computed in the period before the perturbation (8 s window).

Using the four categories of features, generalized linear models (GLM) were built to detect impairments in responses to perturbations. Given that responses would be different based on the perturbation, a model was built for each perturbation. The two phases of model building were feature selection, followed by parameter fitting, with data held out at each phase. Features were selected based on their ability to discriminate between the two subject populations effectively, as assessed using Cohen’s *d* values. Principal components were computed using the discriminative features and a GLM was fit to the components. The performance of the models were evaluated on data held out during the model building phase.

**Figure 3:**
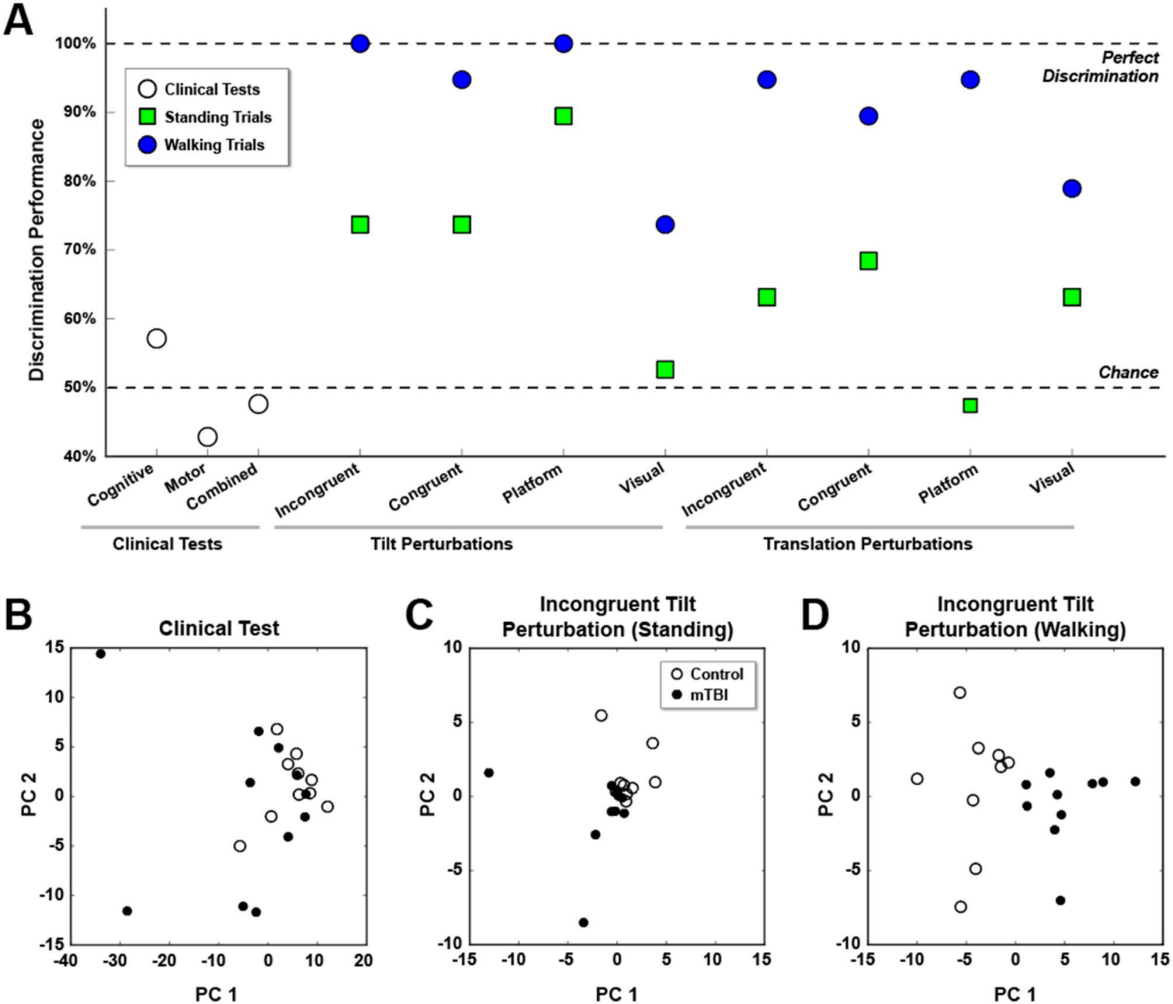
Model accuracy as a function of perturbation type. (**A**) Detection of mTBI using sensorimotor perturbations outperforms current clinical assessments. For each perturbation type, a model was built to detect whether an individual was a healthy control subject or had incurred an mTBI. Data shows the average classification accuracy of the models on data that was held-out during model building. Moreover, separate models were built using data from walking (blue circles) and standing (green squares) trials. As compared to the clinical tests (open circles), the models generally have higher classification accuracy, especially given data from trials in which subjects were walking rather than just standing. In the bottom panels, the principal component space is visualized to illustrate the capability of separation. Separability is poor using (**B**) clinical tests, but is improved when using (**C**) perturbations while the subjects remain standing. The best capability for discrimination is provided by (**D**) perturbations applied during walking, wherein healthy control subjects (open circles) are clearly distinguished from subjects with an mTBI (closed circles).

Results indicate that, as compared to standard clinical tests, features derived during sensorimotor perturbation are effective at detecting whether an individual reported lingering balance impairments from an mTBI (Fig. 3). The discriminative capabilities of these models were greatly improved if the perturbations were applied during periods of walking (average ≈ 0.90) as opposed to periods when the subjects were standing (average ≈ 0.65). Unlike the motor-targeted clinical tests (BERG, DGI, and HiMAT) that were insensitive and non-specific, the sensorimotor perturbations elicited nuanced motor changes enabling finer comparisons and improving detection performance.

The perturbations that involved multi-sensory conflicts (i.e., incongruent translation and rotation) yielded the highest performance at discriminating the subjects. The platform-only movements also showed high capability for discrimination, even in the condition where subjects were standing and not walking. While visual-only perturbations provided less discriminative capability than the other types of perturbations, they still provided more capability than the standard battery of clinical tests. The subject population was diverse in age and in the time since injury (see Table S2) and yet, the model performance was not dependent on the demographics. Linear regression analysis showed that model performance was not a function of subject age (Standing Conditions: β = − 0.002, p = 0.61; Walking Conditions: β = 0.001, p = 0.65) or the time since injury (Standing Conditions: β = − 0.001, p = 0.87; Walking Conditions: β = − 0.001, p = 0.34).

### Number of trials required to maximize model accuracy

**Figure 4:**
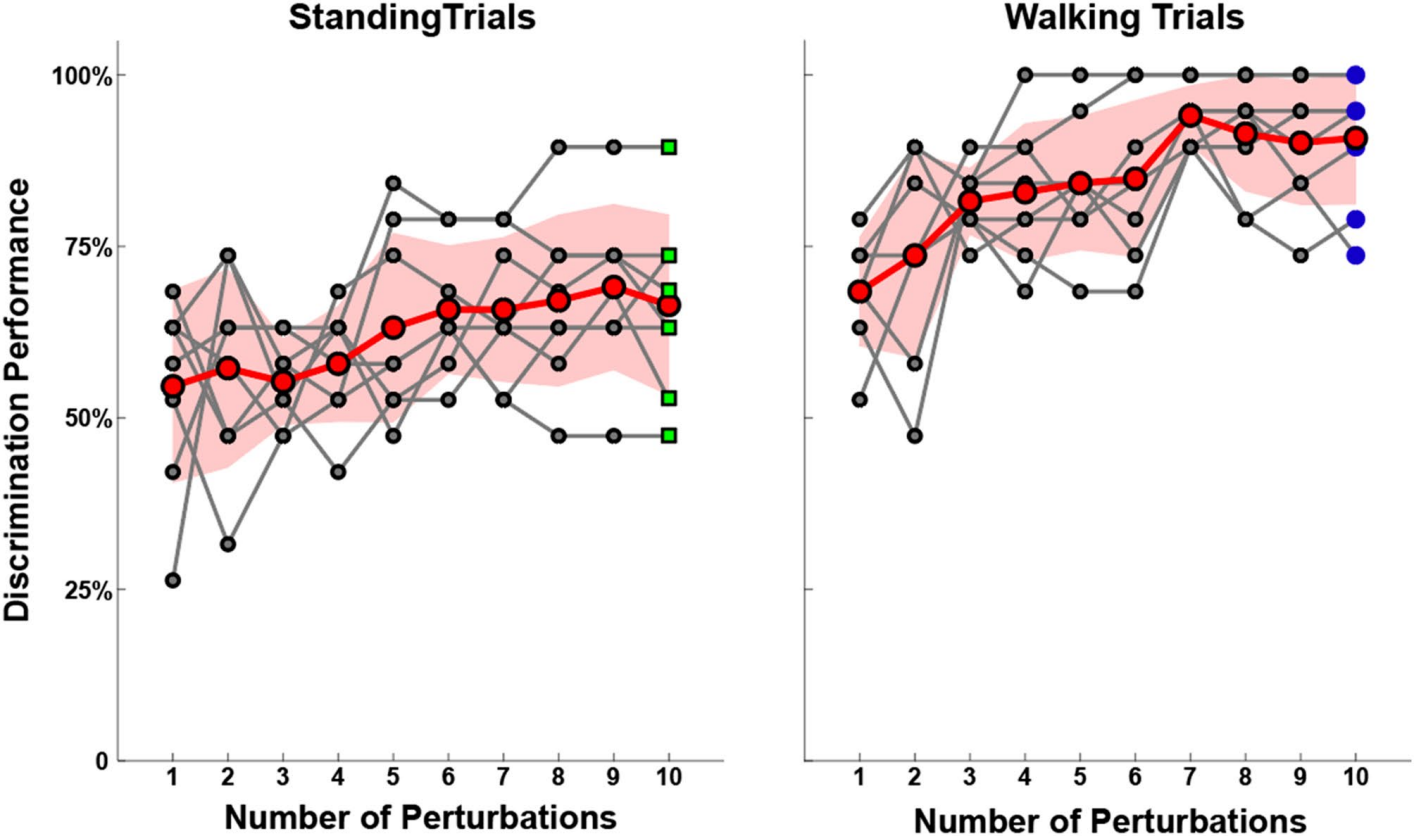
Model accuracy as a function of number of perturbations performed. To produce these results, data from perturbation trials were iteratively added, in the order of presentation in the experiment. Models were built using just the number of perturbations noted on the abscissa. Data points in grey represent the results from each of the eight perturbation types while data points in red show the mean (± sd) across all conditions. The data points that use data from all ten perturbations are exactly those shown in Fig. 3 and are denoted by shape and color as such. Detection of mTBI improves with an increasing quantity of perturbations, but more so for walking trials than standing trials. While the detection rate stagnated after a couple iterations for standing trials (left), the performance continued to increase through iterations for walking trials (right).

An individual’s response to a given perturbation may be variable due to natural human variability or potentially the severity of the injury. To characterize the extent of intra-subject variability as well as the amount of data needed to discriminate between populations, models were built iteratively by incrementally adding data from a subject. Since every subject experienced each perturbation ten times, ten models were built wherein each subsequent model had data from the subsequent perturbation. For example, the first set of models was built using data collected from only the first instance of the perturbation applied to the subjects. The second set of models was built with only the first and second instances of the perturbations. Extending this method, the full set of data is shown in Fig. 4. Note that the performance values at iteration 10 are identical to those shown in Fig. 3.

### Contributions of feature types based on perturbation

The rise in model performance with increasing inclusion of trials was more pronounced for the walking trials as compared to the standing trials. Conversely, while the variability in the features derived during walking contributes to the poor model performance with just one or two trials, the increased data mitigates such variability and permits teasing apart differences in the populations. Out of the sixteen conditions shown in Fig. 4, only two conditions (Standing: Congruent-Tilts, Walking: Visual-Translation) showed worse performance after all the data was included. Further investigation, not possible with these data, would be needed to determine whether detection accuracy could be further increased with more data in the walking conditions.

**Figure 5:**
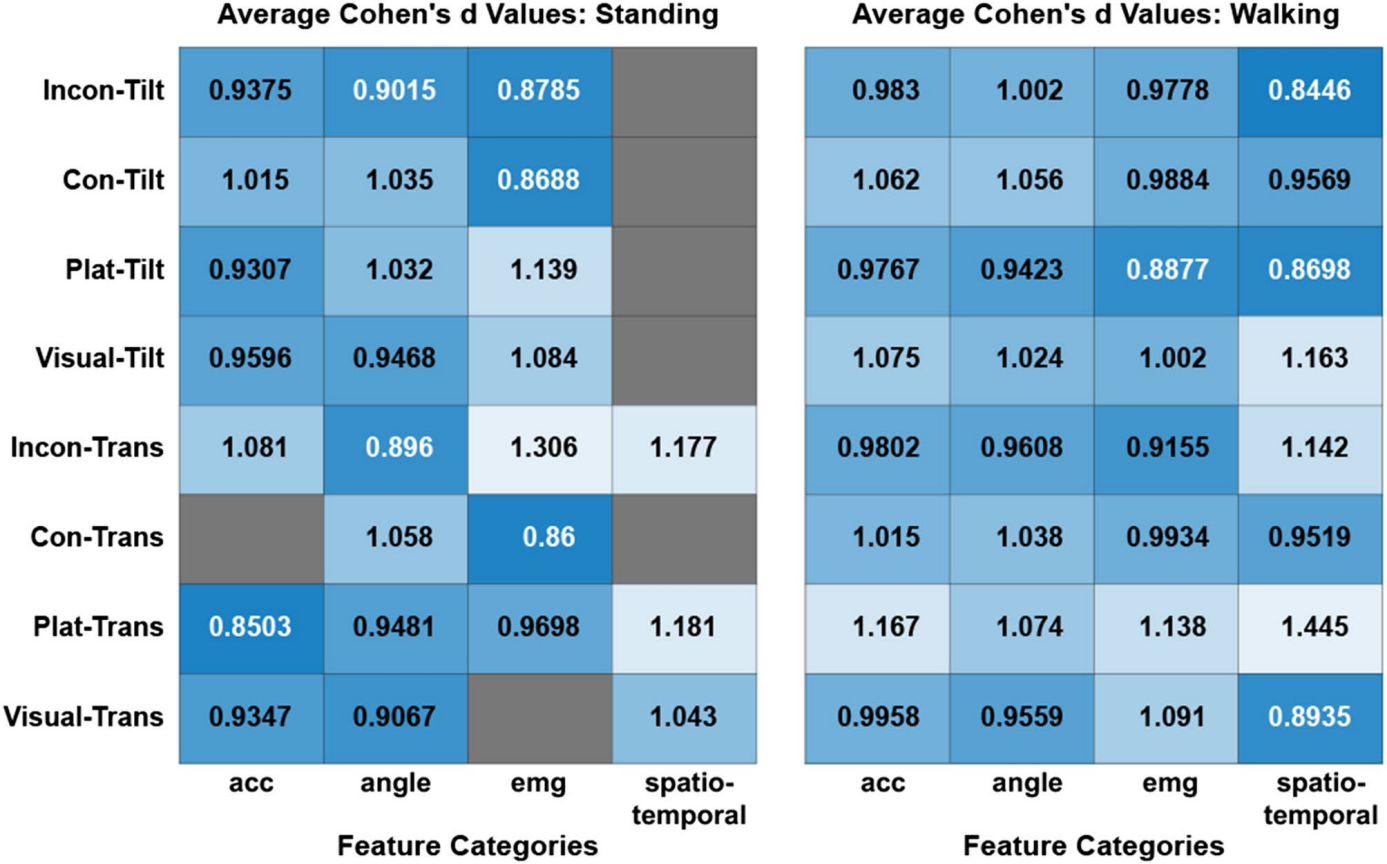
Contributions of feature types to model discrimination capability. Of the features included in the models, the median Cohen’s *d* values are shown for standing (left) and walking (right) trials. Blank cells indicate feature categories without any substantial contribution to that perturbation model. Darker shades of colors indicate cells with lower values. The vertical axis corresponds to perturbation types.

Each perturbation challenges the subjects’ balance in different ways. Therefore, the models that discern between the subject populations would use different features of static and dynamic balance. To evaluate the contribution of each feature category to the detection of mTBI, the median Cohen’s *d* value for each perturbation model is shown in Fig. 5. Only features that were selected for model building, i.e., those with Cohen’s d values greater than 0.8, are represented. All categories of features were informative in detecting mTBI during the walking trials, but some feature categories were uninformative in the standing condition (Fig. 4; black cells).

### Patterns of changes within feature categories

**Figure 6:**
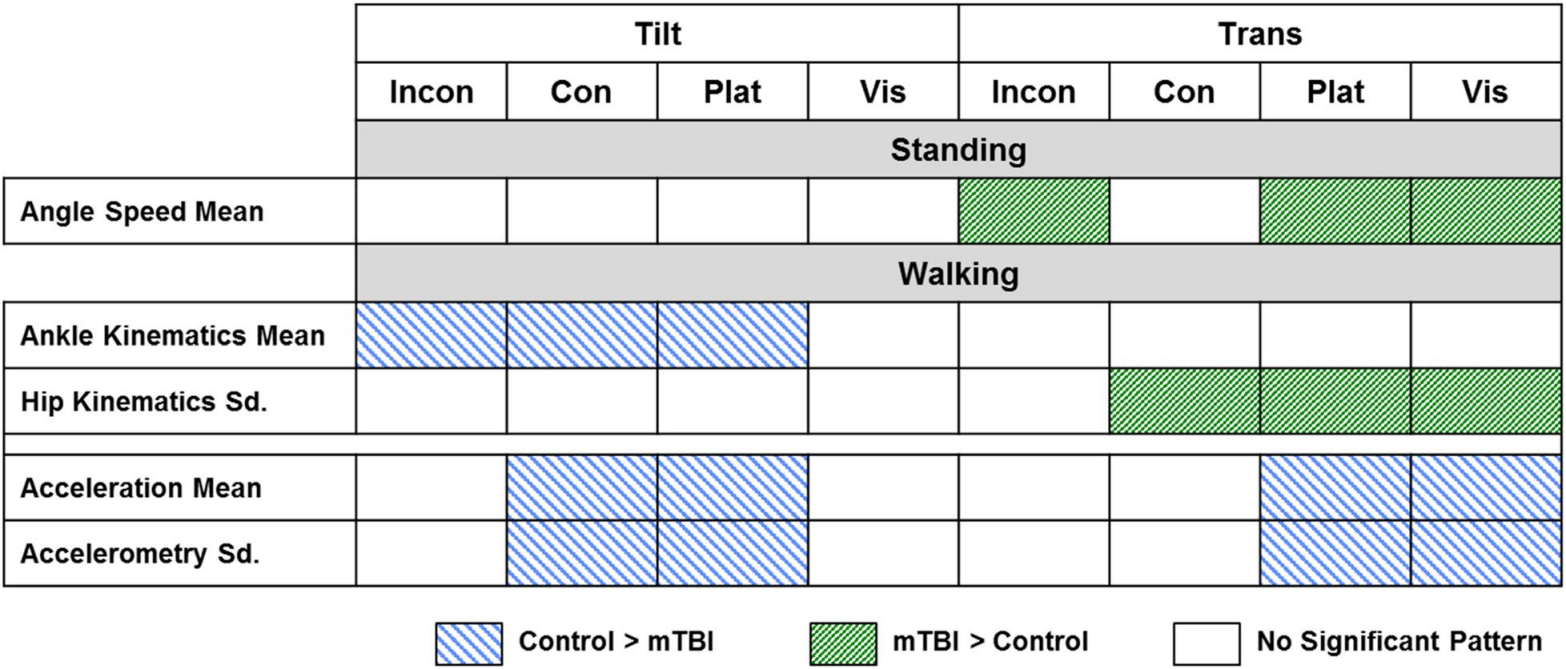
Patterns of feature changes in response to perturbations. If a significant pattern of difference between mTBI and control subjects was found amongst the features, the box for the corresponding feature category and perturbation was colored.

Features shown to be discriminative of the two populations can provide clinicians with insight into how patients with mTBIs react during provocative perturbations. For each feature, which is a difference of the values computed before and after a perturbation, a comparison was made between the averages for mTBI and control subjects. Specifically, the features were labeled as either having larger values, on average, for mTBI subjects or having larger values, on average, for the control subjects. A two-sided binomial test was conducted using these labels for all of the features, as well as the features in-aggregate, for each of the four feature categories. The results from these tests are shown in Fig. 6. A significant result indicated that the features used in the predictive model reflected systematic patterns of differences between mTBI and control subject behaviors.

Differences in groups of subjects manifested themselves as patterns of modification in feature categories for many of the walking and standing perturbations. In standing conditions, mTBI subjects show greater speeds in joint angle changes during incongruent-, platform-, and visual- translational perturbations, reflecting that mTBI subjects might have to go through a series of faster movements to regain balance, while control subjects can quickly come back to their pre-perturbation state without needing extra movements. During walking, ankle and hip kinematics drive many of the differences between control and mTBI subjects. Control subjects respond to platform, incongruent, and congruent-tilt perturbations by adjusting their ankle flexion, as opposed to mTBI subjects who show little change post-perturbation. For the congruent, platform, and visual-translation perturbations, mTBI subjects display greater variability in their hip kinematics, moving their hips in an attempt to maintain gait. It should be noted that all subjects showed normal lower-limb range of motion in clinical assessment. Subjects with an mTBI are not relying on ankle strategies to regain balance in tilt and translation perturbation conditions, suggesting they feel the effects of the perturbation more than control subjects do. Healthy control subjects can rely on increased and more variable acceleration patterns to regain their balance during translational and tilt perturbations, but mTBI subjects appear to maintain a stiffer lower body, in an attempt to maintain balance after a perturbation.

## Discussion

As compared to traditional clinical tests, provocative sensorimotor perturbations are effective at revealing the presence of balance impairments due to mTBI. To discriminate between the healthy controls and the subjects who have had an mTBI, features related to gait and balance were used. Specifically, the changes in features in response to sensorimotor perturbations were informative of the individual’s impairments. Of these perturbations, rotations of the platform, independently or in conjunction with any rotation of the visual scene, yielded a high degree of discriminative capability. Further, models produced more accurate discriminations during trials where perturbations were applied during walking as opposed to during standing. During standing trials, the kinematic features were most discriminative, while the accelerometry features were more so during the walking trials. These results indicate that standard clinical assessments are missing important components within gait, and thus, the clinical tests could be improved by integrating kinematic and accelerometry-based information.

Better discrimination was observed if perturbations were applied while subjects were walking as opposed to standing. This observation might be attributed to two factors. First, during walking, the problem of dynamic balance is more challenging than static balance (i.e., standing). Correction in response to unpredictable perturbations requires better sensorimotor control while walking than standing^21^. Second, features of the gait cycle, as compared to features of standing only, provide more options for analysis, as there are a greater overall number of features to consider in the walking condition. Features computed during standing-only conditions could also be computed during walking conditions, but features related to the gait cycle could not be computed during standing only conditions. It is important to note, however, that the same feature type, computed in the standing or walking conditions, would have different interpretations.

Model fitting procedures were performed so as to promote generalizability. Specifically, individual subjects were held out during model building, and only the accuracy of predicting on the held out-subject was reported. Yet, further investigation is needed on larger populations of subjects to assess the capacity of these models at detecting mTBI. With the current subject population, the details of their specific deficits (e.g., vestibular, visual, or proprioceptive) were unknown. The perturbations were designed to deliver specific sensorimotor conflicts to help diagnose the phenotype of impairment. A challenge with the present participant population was the lack of identified phenotypes. Future modeling efforts, informed by the nature of the subjects’ deficits, would help in validating the current methodology.

Variability is inherent in gait, and therefore variability is to be expected in the response to perturbations. To characterize the extent that variability would influence the ability to discriminate between the two subject populations, the experimental protocol had ten instances of each perturbation. The results indicate that including data from multiple perturbations increased the capability of models to discriminate between the populations. However, the data from walking trials predicted significantly above chance even with data from just a single perturbation. The implications for developing new clinical tests, therefore, are that multiple iterations are required.

In clinical practice, hip and ankle strategies have traditionally been used to detect balance impairments^22^. Hip strategies are typically employed during fast or large amplitude perturbations, and are seen more prevalently in clinical populations^23^. Our approach of transforming features into principal components served to highlight the idea that that combining multiple features from walking perturbations captured subtleties of gait and balance that could not be captured through use of individual features in classification or through use of standing features. Specifically, the use of hip strategies versus the use of ankle strategies by mTBI subjects suggests that the translation perturbations had a greater effect than they did on control subjects, a distinction that was not captured through standard clinical balance measures. Using the data driven approach enabled by the CAREN system and our analysis, we have the ability to explore different combinations of joints in balance strategies that complement and go beyond the traditional grouping of hip and ankle strategies.

By testing a superset of the technologies and algorithms in the laboratory setting, just the most informative aspects of the system or procedure can be translated to the clinic. Prototyping in the laboratory before translating to the clinic reduces the burden on the clinician while providing access to the latest technological advances. In accordance with our goal of improving upon existing clinical tests, the current standards of care serve as an important benchmark for laboratory research. New technology (e.g., head mounted virtual reality displays) can serve to either augment transitional clinical tests or to provide insight into the development of new clinical tests. In both cases, the prototype developed in immersive virtual environments address the dimensions of traditional clinical tests that are currently not being evaluated.

The heterogeneity of the subject population is both a limitation and a strength of this study. All the subjects in the mTBI group had sustained a physical trauma to the head and sought medical attention for it (see “Subject recruitment”). However, there is variability in the type of injury, time since injury, and whether subjects continue to see a physician for lingering symptoms. In the present study, the heterogeneity is embraced because, from a clinical perspective, the population is truly varied, which presents challenges that clinicians must face when diagnosing and treating individuals who have sustained an mTBI. Despite this heterogeneity, the present results indicate that there may be a link between mTBI and subtle changes in balance strategy that can be detected through precise measurement during sensorimotor provocative tests.

These results do not indicate a test that is ready for clinical use, including as a diagnostic, for mTBI or any other neurological condition. Rather, the present study stands as an exploratory proof-of-concept, implying that detailed quantification of static and dynamic balance characteristics, especially when elicited with sensorimotor provocative tests, may provide a window into mild neurological pathologies that are clinically challenging to assess, consistent with the primary goal of this study. Further work is needed to demonstrate the validity of this approach, in order to establish whether it is appropriate, from a clinical or research perspective, as the basis for a test—e.g., a diagnostic. Validity could be established by employing our approach in a study with a more homogenous sample in which the presence and phenotypes of injury are known and can be firmly determined using gold-standard methods (e.g., biomarkers, brain imaging, and physiological measures). Further, the specificity of the approach can be validated by discriminating between mTBI and other clinical neuromotor dysfunction.

The present work has focused primarily focused on manipulating sensory feedback, perceptual processing and sensorimotor integration by perturbing the motion base and the visual scene in the CAREN system immersive environment. However, our experiments have not yet explored the full range of sensorimotor provocative testing possible. For example, the CAREN system is equipped with a split-belt treadmill, which has been demonstrated as a useful tool for investigations into motor planning, especially through motor adaptation experiments^24,25^. Similarly, the additional degrees of freedom available in the motion base may allow for variable surface compliance and manipulation of tactile cues, an approach which has been demonstrated by Artemiadis and colleagues^26^ with applications to stroke patients. It may also be possible to take inspiration from, or even augment, existing CAREN systems to implement more direct manipulation of limbs for proprioceptive perturbation, perhaps with the integration of use of cable-driven and/or exoskeleton systems (e.g., Jin et al.^27^). Incorporating these additional techniques and associated experimental paradigms would be of great interest, as they have been utilized in the context of a broader range of neurological deficits (e.g., cerebellar damage, and motor planning deficits), and would therefore complement the present techniques. Indeed, the present subjects also participated in motor adaptation experiments as part of this same protocol, and immediate future work will involve analyzing those data, and also incorporating mechanistic modeling that may allow the association of these motor behaviors with specific impairments.

## Materials and methods

### Subject recruitment

Subjects between the ages of 18 to 80 years were recruited as part of a study on static and dynamic balance assessment in the context of mTBI. A subject was grouped into the mTBI category according to the American Congress of Rehabilitation Medicine (ACRM) definition of: “a traumatically induced physiological disruption of brain function, as manifested by at least one of the following: (1) any period of loss of consciousness; (2) any loss of memory for events immediately before or after the accident; (3) any alteration in mental state at the time of the accident (e.g., feeling dazed, disoriented, or confused); and (4) focal neurological deficit(s) that may or may not be transient; but where the severity of the injury does not exceed the following: loss of consciousness of approximately 30 min or less; after 30 min, an initial Glasgow Coma Scale (GCS) of 13–15; and posttraumatic amnesia (PTA) not greater than 24 h”^28^. Subjects with neurological injury less than 1 week prior to the day of the experiment were excluded. Additionally, subjects that had other major neurological or psychiatric diseases were excluded. At the time of the experiment, none of the subjects had active changes in medication, indicating a stable regime during evaluation. The procedures were approved by MIT's Committee on the Use of Humans as Experimental Subjects (COUHES) Institutional Review Board. All experiments were performed in accordance with the approved guidelines and regulations. All subjects signed an informed consent prior to participation in the study.

### Experimental protocol

Experiments were conducted at the Massachusetts Institute of Technology Lincoln Laboratory's (MIT LL) Sensorimotor Technology Realization in Immersive Virtual Environments (STRIVE) Center. Clinical tests were administered on all subjects by a trained physical therapist to assess the cognitive and motor functions of the subjects. Subsequently, the CAREN-based experimental protocol was performed. The clinical tests lasted approximately 1.5 h and the CAREN-based segment also lasted 2 h.

### Clinical assessments

As a basic cognitive screen and to assess visual attention, the Montreal Cognitive Assessment (MoCA) and the Cognitive Linguistic Quick Test (CLQT) were used^29–31^. To determine subjective confidence in performing various ambulatory activities without falling or experience unsteadiness, the Activities-Specific Balance Confidence Scale (ABC) survey was conducted^32,33^. The Rivermead Post-Concussion Symptoms Questionnaire was administered to determine post-concussion symptom presence and severity^34^. To assess motor, sensory, balance and vestibular function, an evaluation of joint range of motion, muscle strength, proprioception, Berg Balance Scale, BESTest, Dynamic Gait Index (DGI) and Dix-Hallpike Maneuver were administered^32,35–38^. Finally, to assess high level mobility, the High-Level Mobility Assessment Tool (HiMat) was used^12^.

### CAREN system

The Computer Assisted Rehabilitation ENvironment (CAREN) system is a commercially available immersive environment system manufactured by Motekforce Link (Amsterdam, Netherlands). The system is designed to study human movement and physiology by providing subjects with a visual and physically immersive, virtual experience. The system includes an instrumented, dual belt treadmill mounted on a six degree of freedom motion base. This combination device is centered in a 7 m diameter dome that doubles as a 360° projection screen to immerse the subject in a virtual reality world. Various physiological sensing systems are integrated into the system. Subjects may interact with this virtual world using one or more of these modalities.

### CAREN-based experimental protocol

The experimental protocol included two baseline blocks (one at the start and one at the end), four reactive perturbation blocks (two standing and two walking), and four repeated perturbation blocks (two standing and two walking). Each block contained multiple trials lasting up to 5 min each. Within a trial, perturbations were delivered with an interstimulus interval of between 8–12 s. In an attempt to prevent subjects from anticipating the next perturbation, the protocol always included a “Shape Task” wherein objects (squares, circles, triangles) of different colors (red, green, blue) were flashed on the screen at a random interval between 8–12 s. Subjects had to say the shape and color as soon as they saw it. Paying attention to the visual shapes task focused the subjects’ attention straight ahead, thereby removing confounds of head rotations away from the screen, which could also cause loss of balance. The Shape task was used as an orienting guide and to keep subjects engaged. It was not designed as a dual task and subjects’ performance was not recorded or scored. Therefore, at the end of each interstimulus interval, either a sensorimotor perturbation was delivered or a shape was presented, never both simultaneously. The ordering of perturbations and colored shapes were randomly determined prior to the start of the study and every subject experienced the same overall ordering of perturbations and colored shapes.

During each of the two baseline blocks, subjects performed two quiet standing trials, performed with eyes open and eyes closed, each 3 min in duration. Subsequently, each subject performed a walking trial at ~1.0 m/s for 3 min in duration. The speed was varied slightly (0.7–1.2 m/s) depending on the subjects’ preference. A subject walked at a given pace for the duration of the study. No perturbations were applied in the baseline tasks.

During the reactive perturbation blocks, subjects performed two trials in which they attempted to stand still and maintain balance while single provocative perturbations were imposed on them. Then, subjects performed two trials in which perturbations were applied as they walked at constant speed. In the first of the walking trials, perturbations were timed to the incidence of left-foot heel strikes. In the second walking trial, the perturbations were timed to the right-foot heel strikes. Presented in this paper are results from just the reactive perturbation trials.

During the adaptation blocks, subjects similarly performed two standing trials and two walking trials but this time each trial was 2 min long and platform-only perturbations were applied continuously for 1 min, starting at 15-s into the trial. To deliver the platform tilt, the platform was rotated and held at that incline for the duration of the perturbation. To deliver the platform translation perturbation, the platform was rapidly translated at the time of each right-foot heel strike. The platform was returned to its original position before the next gait cycle in order to be translated again.

### Physiological and biomechanical data collected

The Vicon motion capture system was used to record the movements of reflective markers, which were placed on anatomical landmarks on the subject as prescribed by the lower-body Plug-in-Gait model. Electromyography (EMG) was collected using the Delsys Trigno wireless system (Natick, Massachusetts, USA). Electrodes were placed on the lower body muscles Medial Gastronemius, Tibialis Anterior, and Vatus Lateralis. Each Delsys sensor had a three-axis accelerometer, which measured the accelerations at those three locations on each leg. Additionally, event information about the perturbations (visual and platform perturbations) were stored using the experimental software, D-Flow, which was used to control the CAREN system.

**Figure 7:**
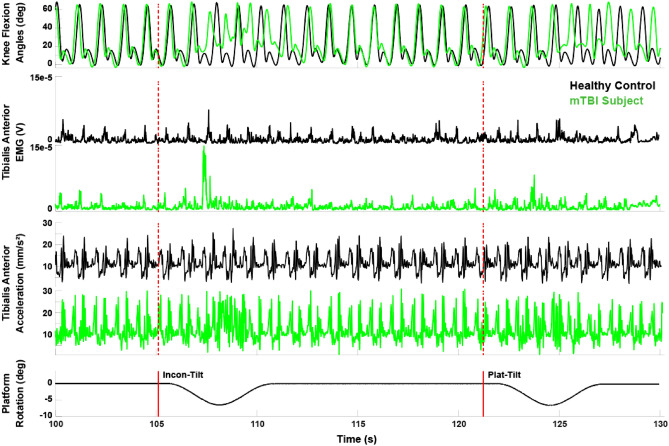
Example data signals collected during experiment. Data from a healthy control (black traces) are plotted against data from a subject with mTBI (green traces). Shown are single examples from the modalities of data collected—kinematic (e.g., left knee flexion angle), EMG (e.g., from left Tibialis Anterior muscle), and accelerometry (e.g., from left Tibialis Anterior). The perturbations, incongruent tilts of platform and visual scene (*Incon-Tilt*) and platform-only tilt (*Plat-Tilt*) impair the balance of the mTBI subject more than the healthy control.

Example signals from each of the modalities are shown in Fig. 7. As a comparison in behavioral performance between subject populations, the figure shows time series of joint angles, EMG activity, and accelerometry traces from a control subject (black) and a subject with mTBI (green). Further depicted are the timings of perturbations (red lines) and the platform rotations. Not shown are the movements of the virtual visual scene projected around the subject in the CAREN system.

This example shows two sequential perturbations and their effect on the subjects’ gait. The first perturbation is an incongruent tilt (“Incon-Tilt”), wherein the platform tilts downward and the visual scene rotates upward. The second perturbation is just a platform tilt downward, similar to the first perturbation, but without the visual scene change. Note the striking differences in gait patterns between the two subjects after each perturbation. In the case of the control subject (black traces), there is little impact on joint kinematics, though a marginal correction is observed through the EMG and accelerometry activity. However, the subject with mTBI (green trace) shows imbalance and corrective behavior in the form of faster steps with less range of motion but higher muscular activation. As a comparison between the two perturbations, the influence that the second perturbation has on the mTBI subject is qualitatively less than the first perturbation even though the platform tilt is the same. Thus, for this mTBI subject, the incongruent sensorimotor information seemed to have a strong impact on balance, whereas the healthy control was unaffected by either perturbation.

### Data pre-processing

Motion of the reflective markers, adhered to the subject as prescribed by the lower body Plug-in-Gait model, were used to calculate the motion of the subject and biomechanical models. Motion capture data was collected at 250 Hz. In cases where markers were dropped from camera view for short periods, the data were automatically interpolated using gap filling techniques built into Nexus. All computation of gap filling and biomechanics was performed using algorithms built into Vicon Nexus (version 2.6.1). Where markers were part of rigid bodies (e.g., hips), the ‘rigid body fill’ was used. In cases where the markers were obscured for less than 20 ms, a quintic spline interpolation was used (‘spline fill’). In cases where the markers were not part of a rigid body but obscured for greater than 20 ms but less than 100 ms, a pattern derived from the closest marker was used to predict the obscured marker movement. Finally, markers obscured for more than 100 ms but less than 250 ms were filled using trajectories estimated based on the kinematic fit of joints (“Kinematic Fit”). It is possible that even with these gap-filling steps, certain gaps in marker positions would not be filled due to long durations of being obscured. In such cases, that window of data for that marker was excluded from further analysis.

The EMG and accelerometry signals were collected at 2000 Hz. The EMG signals were bandpass filtered from 20 to 250 Hz with a second order IIR filter, followed by rectification, and a median filter with a 25 ms sliding window. Each axis of the accelerometry data was bandpass filtered from 0.01 to 30 Hz with a second order IIR filter.

To compute gait cycles, the heel strikes and toe offs were calculated for each leg from the positions of markers on the feet. The relative velocity of the feet and the hips were used to determine the point of maximal forward position of the feet relative to the hips (i.e., heel strike) and maximal backward position of the feet relative to the hips (i.e., toe offs)^39^.

### Feature calculation

Four categories of features were calculated to characterize the behavior of gait and balance (Table 1). Spatiotemporal features such as stride length, width, durations, and ratios were computed based on the gait cycles. The positions and speeds of movement correspond to the movement of the center of mass of the subject, relative to the center of the treadmill, through time. The kinematic joint angles included the angles of the hips, knees, and ankles of each leg in three degrees of freedom (flexion, abduction, and rotation). The ranges of motion were computed as the difference in the 5th and 95th percentile of angles. The mid-stance and mid-swing were calculated as the midpoints in time between the heel strikes and toe offs. For both the EMG and accelerations, the average of the data, “Values”, were computed within the windows of interest or at specific points along the gait cycle. Specifically for the accelerations, the features were computed in each of the three axes. For all the feature types, the means and standard deviations were computed and used as independent features. For all features related to the gait cycle (GC), the feature values were computed within a gait cycle. For trials in which there was no gait (NG), the features were computed as means and standard deviations across the window of interest.

**Table 1:** Features used to build discriminative models.

Spatiotemporal	Kinematic	EMG	Accelerations
Stride length_GC_	Joint angles_NG,GC_	Values_NG,GC_	Values_NG,GC_
Stride width_GC_	Angle range of motion_NG,GC`_	Range_NG,GC_	Range_NG,GC_
Stride duration_GC_	Angle speed_NG,GC_	Value at heel strike_GC_	Value at heel strike_GC_
Swing duration_GC_	Angle at heel strike_GC_	Value at Toe off_GC_	Value at Toe off_GC_
Double support ratio_GC_	Angle at Toe off_GC_	Value at midswing_GC_	Value at midswing_GC_
Cadence_GC_	Angle at mid-swing_GC_	Value at midstance_GC_	Value at midstance_GC_
Position and speed in 3D_NG,GC_	Angle at mid-stance_GC_		

Within each of the four categories of features, means and standard deviations of the listed features were calculated. All features could be computed within gait cycles (GC) but some features could not be computed if there was no gait (NG). The labels of GC and NG denote features that were computed within a gait cycle or irrespective of the gait cycle.

**Figure 8:**
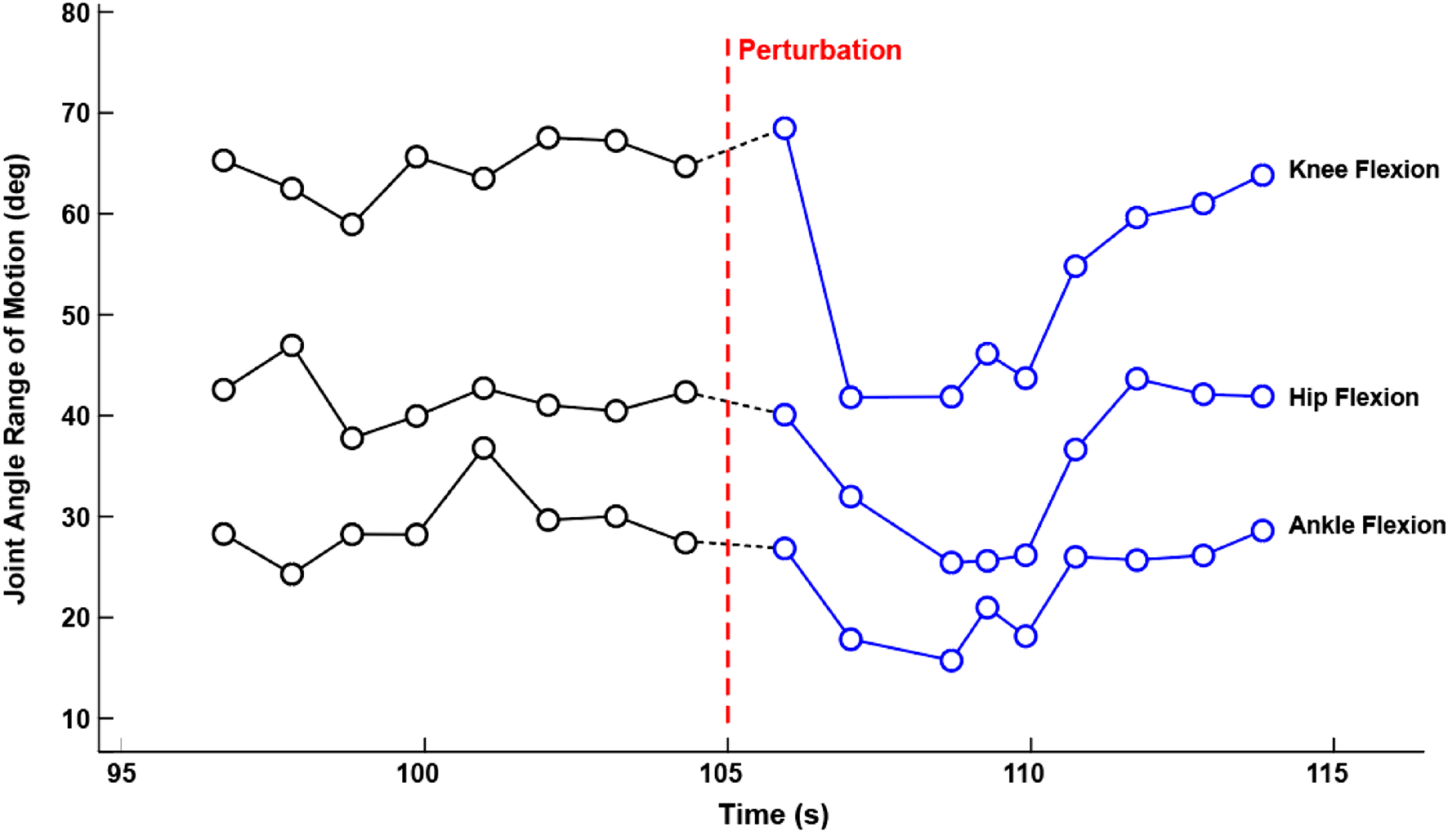
Example feature changes in response to a perturbation. To isolate the individualized response of the perturbation, the mean of each feature, calculated before the perturbation (black), was subtracted from the mean of that feature calculated after the perturbation (blue). In this example, three features were calculated corresponding to the change observed in the ranges of motion of the hip, knee, and ankle flexions.

Recall that our hypothesis is that the sensorimotor perturbations would elicit greater changes in postural adjustments for subjects with mTBI as compared to the healthy controls. For the mTBI subject shown in Fig. 7, the corresponding example of features are shown in Fig. 8. The feature category in this example is the joint angle range of motion. Each data point corresponds to the range of motion through a single gait cycle. The ranges of motion for the knee, hip, and ankle flexion are steady prior to the perturbation (black), but are greatly reduced in response to the perturbation (blue). Not only do the ranges in motion decrease, it can be inferred that that the variability in cadence also increases. Soon after the perturbation end (at 110 s), the gait pattern returns to that observed prior to the perturbation.

To isolate the influence of the perturbation on changes in balance while minimizing natural variability in individual differences, the feature categories listed in Table 1 were computed in the period before the perturbation (8 s window) and in the period after the perturbation (5 s window). The post-perturbation values were normalized through subtraction of the pre-perturbation values. In the case of calculating means, the mean post-perturbation values were subtracted from the mean pre-perturbation values. In the case of calculating standard deviations, the standard deviation of the post-perturbation values were subtracted from the standard deviations of the pre-perturbation values. Those differences in values, calculated for all categories listed in Table 1, formed the set of features used in subsequent modeling.

### Feature selection and model building

For each perturbation type, a model was built to detect mTBI using balance-relevant features. Each of the eight models were built in two phases—feature selection, then parameter fitting. To select the features, the Cohen’s d effect size was computed for each feature to assess its ability to separate the healthy controls from the subjects with mTBI. Features with effect sizes above 0.8, indicating “large” effect sizes (Cohen, 1988), were kept for the next phase, while the rest were excluded. On average, there were 115 features included for each model. To fit the parameters of the model, the feature array was reduced in dimension using principal component (PC) analysis and PCs were kept such that 95% of the variance was explained in the data. A logistic regression was fit using the PCs as regressors and mTBI labels as the outcome. When selecting features and building a model, a single subject’s data was held out and the model performance was evaluated on that held out data. Extending the process, each subject’s data was held out and models were built and tested 19 times corresponding to the 19 subjects in the study. The overall performance of this method is reported as an average in model performance across all held out subjects.

## Supplementary Information

Supplementary Information.
