# Supplementary material for: Sensorimotor conflict tests in an immersive virtual environment reveal subclinical impairments in mild traumatic brain injury
Source: Sci Rep. 2020 Sep 8;10:14773. doi: 10.1038/s41598-020-71611-9 (PMC7479615; doi:10.1038/s41598-020-71611-9)

Title: Sensorimotor Conflict Tests in an Immersive Virtual Environment Reveal Subclinical Impairments in Mild Traumatic Brain Injury

**Authors:**

Hrishikesh M. Rao^1*^, Tanya Talkar^1,2^, Gregory Ciccarelli^1^, Michael Nolan^1^, Anne O’Brien^3^, Gloria Vergara-Diaz^3,4^, Delsey Sherrill^1^, Ross Zafonte^4,5^, Jeffrey S. Palmer^1^, Thomas F. Quatieri^1,2^, Ryan J. McKindles^1^, Paolo Bonato^3,4^, Adam C. Lammert^6^

**Affiliations:**

^1^Human Health & Performance Systems, MIT Lincoln Laboratory, Lexington, MA, USA

^2^Speech and Hearing Bioscience and Technology, Harvard Medical School, Boston, MA, USA

^3^Motion Analysis Laboratory Spaulding Rehabilitation Hospital, Boston, MA, USA

^4^Department of Physical Medicine and Rehabilitation, Spaulding Rehabilitation Hospital, Massachusetts General Hospital, Boston, MA, USA

^5^Brigham and Women’s Hospital, Harvard Medical School, Boston, MA, USA

^6^Department of Biomedical Engineering, Worcester Polytechnic Institute, Worcester, MA, USA

*Hrishikesh.Rao@LL.mit.edu

**Supplementary Material**

For each subject scores from standard clinical tests were collected in addition to basic demographics. The heterogeneity of the mTBI subject population was characterized by documenting the time elapsed since injury (at the time of administering informed consent for this study), the number of injuries subjects have had, and whether they are still actively seeking medical treatment for the injury. In the case where subjects had experienced multiple injuries, the time since the most recent injury is reported. Details for each subject are shown in Table S1. The Rivermead Questionnaire (RPQ), which contains questions specific to the experiences associated with a concussion, was not asked of the healthy control subjects. The RPQ and the Activity Balance Confidence Scale (ABC) are not strictly motor or cognitive tests and therefore are shown here, but not plotted in Fig. 2. There is a statistical difference in the ABC scores between the healthy controls and the mTBI subjects (Mann-Whitney, Z = 3.711, *p* = 0.016).

**Table S1. Subject demographics.** For the summary statistics reported in the body of the methods section and visualized in Fig. 2, itemized demographics and clinical assessments are reported for each subject.


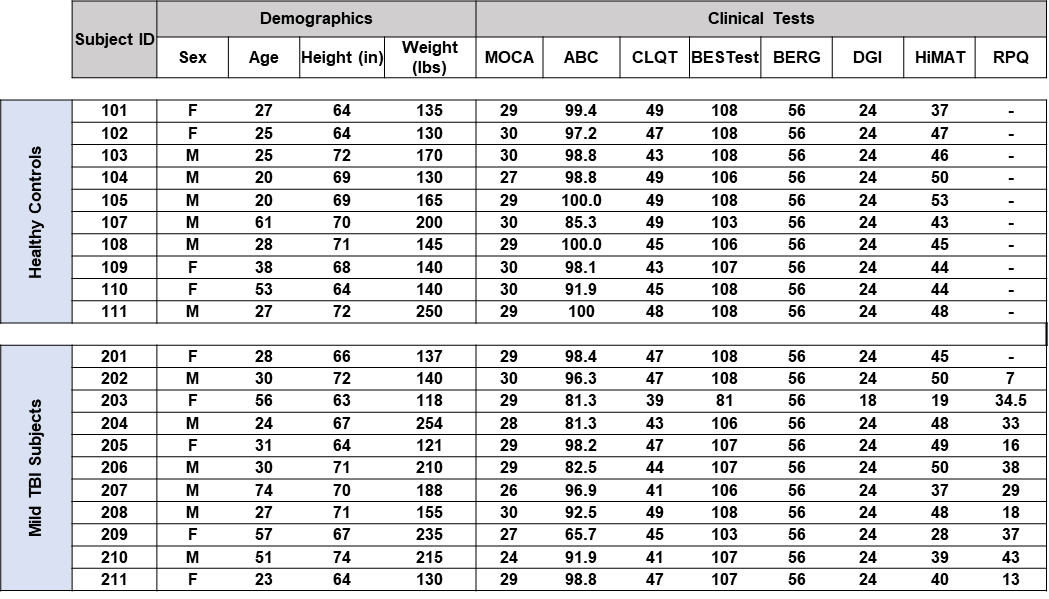


The control subjects were recruited to best match the demographics of the mTBI population. To compare the resulting populations, Wilcoxon Rank Sum *t*-tests were performed for the sexes, ages, heights, and weights, between the control and mTBI subjects. There were no statistical differences in sex (p = 0.69), age (p = 0.23), weight (p = 0.80), height (p = 0.89).

**Table S2. Injury Information.** For each subject in the mTBI group, information is provided about their specific injuries. For individuals that had multiple TBIs, description of the most recent injury is reported.


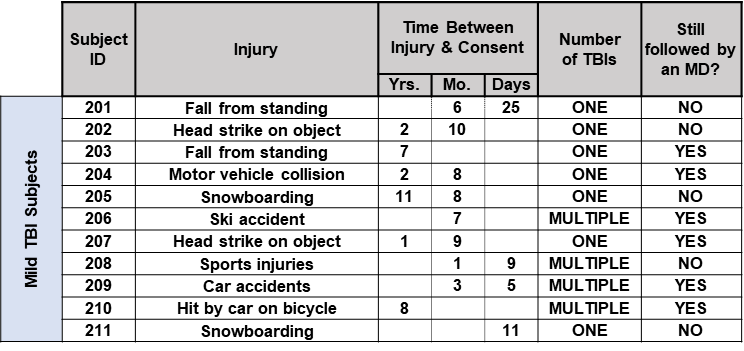


All subjects grouped into the mTBI category had sustained a blunt trauma-induced brain injury (Table S2). The population was diverse in terms of their time since injury and whether they were being followed by a physiatrist for lingering symptoms. The heterogeneity of this medical population is a challenge clinicians face in making diagnoses and prescribing treatment based on symptoms.

In Table S3, the classification accuracy is detailed for each subject and perturbation type. Correctly labeled classifications are shown in green. Two subjects (101 and 202) were not analyzed due to a data collection error (see *Subject Demographics*). The aggregate performances of the models, for each perturbation type, are shown in the bottom row and these correspond to the results illustrated in Fig. 3. This detailed view shows that the inaccuracies of classification are not related to a single subject and also not a function of the time since injury, number of TBIs, or the fact that certain subjects are actively engaged in medical treatment (cf., Table S2).

**Table S3. Detailed classification performance.** Results of the classification accuracy are broken down by subject, perturbation type, and standing or walking trial type. The label of “0” indicates that a prediction of “healthy control” was made and conversely, the label of “1” denotes the prediction of an mTBI subject. For ease of visualization, correctly classified labels are colored in green. Due to a technical glitch, data from two subjects (first healthy control subject and second mTBI subject) could not be processed and were excluded from any analysis.


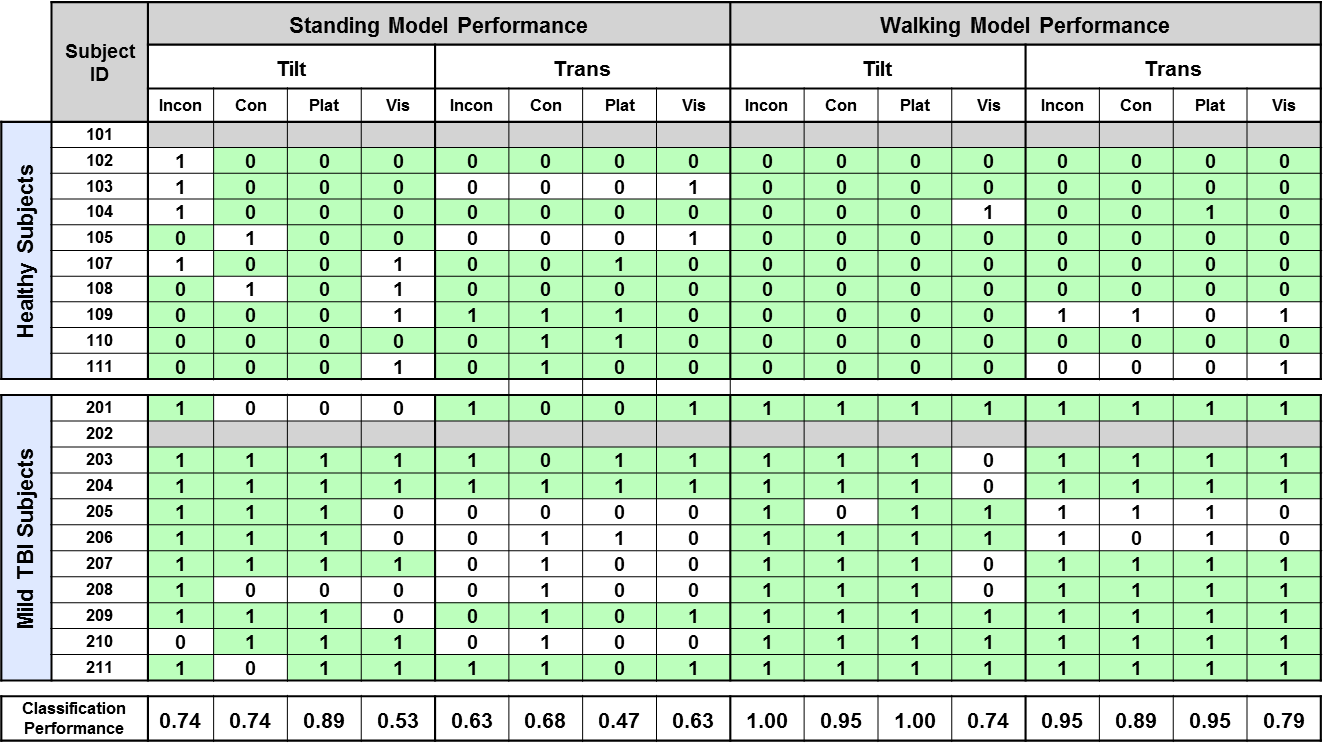

Supplement: Supplementary file 1 — Supplementary Information. [file 41598_2020_71611_MOESM1_ESM.docx]
